# Blur Kernel Estimation and Non-Blind Super-Resolution for Power Equipment Infrared Images by Compressed Sensing and Adaptive Regularization

**DOI:** 10.3390/s21144820

**Published:** 2021-07-14

**Authors:** Hongshan Zhao, Bingcong Liu, Lingjie Wang

**Affiliations:** School of Electrical & Electronic Engineering, North China Electric Power University, Baoding 071003, China; sas_0@ncepu.edu.cn (H.Z.); 2192213093@ncepu.edu.cn (L.W.)

**Keywords:** power equipment, infrared image, blur kernel estimation, non-blind super-resolution, compressed sensing

## Abstract

Infrared sensing technology is more and more widely used in the construction of power Internet of Things. However, due to cost constraints, it is difficult to achieve the large-scale installation of high-precision infrared sensors. Therefore, we propose a blind super-resolution method for infrared images of power equipment to improve the imaging quality of low-cost infrared sensors. If the blur kernel estimation and non-blind super-resolution are performed at the same time, it is easy to produce sub-optimal results, so we chose to divide the blind super-resolution into two parts. First, we propose a blur kernel estimation method based on compressed sensing theory, which accurately estimates the blur kernel through low-resolution images. After estimating the blur kernel, we propose an adaptive regularization non-blind super-resolution method to achieve the high-quality reconstruction of high-resolution infrared images. According to the final experimental demonstration, the blind super-resolution method we proposed can effectively reconstruct low-resolution infrared images of power equipment. The reconstructed image has richer details and better visual effects, which can provide better conditions for the infrared diagnosis of the power system.

## 1. Introduction

The concept of the Internet of Things is proposed, and the era of the Internet of Everything is coming. One of the keys to building the power Internet of Things is to realize online monitoring and analysis and evaluation of the operating status of various power equipment. Among various monitoring technologies, infrared monitoring technology has the characteristics of being long distance and non-contact and having high levels of accuracy and speed [1,2]. The extensive and effective installation of infrared sensors will be one of the key issues that need to be solved in the construction of the power Internet of Things. However, due to the limitation of equipment installation cost, data transmission, and storage capacity, it is obviously difficult to achieve the accuracy of mainstream infrared imagers at this stage using the online monitoring infrared sensor that can be installed on a large scale. Therefore, it is necessary to process infrared images collected by low-precision infrared sensors through background algorithms to enhance their visual effects and enrich their connotative information. The super-resolution technology that has emerged in recent years provides new ideas for solving this problem.

Super-resolution (SR) aims to reconstruct a high-quality image X from its degraded measurement Y [3]. SR is a typical ill-posed inverse problem, and it can be generally modelled as

(1) $$Y=\;\left(k⊗X\right)↓$$

where k is a blur kernel, ⊗ denotes the convolution operator, and ↓ the down-sampling operator. According to the number of input images, SR technology is divided into single-image super-resolution (SISR) technology and multi-frame image super-resolution (MISR) technology. Due to data storage, transmission pressure and other issues, the current power industry does not have the conditions to adopt MISR technology, so we focus on the SISR technology.

The SISR method can be divided into three categories according to the principle. The first type is interpolation methods, which are often simple and easy to implement, but usually excessively smooth the high-frequency details of the image, resulting in poor visual quality of the reconstructed image [4,5,6]. The second type of method is a learning-based method, which learns the correspondence between low-resolution (LR) and high-resolution (HR) image blocks from a given training sample [7,8,9,10]. This results in the effectiveness of the algorithm highly dependent on the selection of training samples. Additionally, when the application conditions change, such as the magnification and degradation information, the model needs to be retrained again which is often accompanied by high computational costs [8]. Therefore, we focus on the third type of methods, namely, reconstruction-based methods. This type of method constructs the model based on the principle of image degradation, and realizes the SR reconstruction of the image by combining prior information in the Bayesian framework or introducing regularization in its inverse problem [11,12,13,14,15,16,17]. Such methods are not limited by samples, are flexible in application and have good reconstruction effects, which are easy to be widely applied in the power grid.

In addition, the SISR method can also be divided into three categories according to the different problems it solves. The first type of method ignores the general blurring in the LR image formation process. These methods consider the LR image to be absolutely clear and only improve its resolution [4,8,11,12]. However, the research of Efrat et al. [18] shows that the influence of blur kernel on the SISR problem is even greater than the influence of the selected SR model. The second type of method is the non-blind SR method, which does not study the solution of the blur kernel, but only focuses on how to reconstruct the HR image from the LR image when the blur kernel is known [13,14,15,16,17]. For example, Glasner et al. [13] used image self-repeatability to reconstruct HR images. Šroubek et al. [14] proved that the degenerate operator can be implemented in the frequency domain and designed a fast-solving algorithm based on this. Dong et al. [15] proposed the concept of sparse coding noise and achieved the goal of image restoration by suppressing sparse coding noise. The third type of method is the blind SR method, which simultaneously solves the problem of blur kernel estimation and HR image reconstruction. However, the joint restoration of blur kernel and HR image is usually difficult, and it is easy to produce sub-optimal reconstruction results [19]. Therefore, there are few studies on blind SR methods [20,21,22,23]. Shao et al. [20] proposed a non-parametric blind SR method based on an adaptive heavy-tail prior. Qian et al. [21] proposed a blind SR restoration method based on frame-by-frame non-parametric blur estimation. Kim et al. [22] proposed a single-image blind SR method with low computational complexity, and Michaeli et al. [23] proposed a blind SR method based on the self-similarity of the spatial structure of image blocks.

In order to improve the quality of SR reconstructed images, to meet the actual needs of the power industry, we propose a blind SR method. Since the joint restoration of the blur kernel and HR image may produce sub-optimal reconstruction results, we chose to estimate the blur kernel first, and then reconstruct the LR infrared image through the non-blind SR method. For the blur kernel estimation, we improved the basic SR model of compressed sensing and introduced the image Extreme Channels Prior to the model; thus, we propose an LR image blur kernel estimation method based on the compressed sensing theory. For the non-blind reconstruction after blur kernel estimation, we propose an adaptive non-blind SR reconstruction algorithm. The algorithm uses adaptive control of the intensity coefficient of the regular term in the reconstruction process to suppress the generation of artifact ringing and improve the quality of the reconstructed image. The final experimental results show that our proposed blind SR reconstruction method for infrared images of power equipment can effectively reconstruct LR infrared images through successive blur kernel estimation and non-blind reconstruction. The reconstructed image has richer details and better visual effects, which can provide better conditions for the infrared diagnosis of the power system.

## 2. Blur Kernel Estimation Method

### 2.1. Basic SR Model of Compressed Sensing

Our blur kernel estimation model is improved from the basic SR model of compressed sensing. So, we first briefly introduce it, and its model is:

(2) $$y=SK\Psi \tilde{x}$$

where y is a one-dimensional vectorized LR image; S is the down-sampling matrix, which is generally generated according to the principle of cubic interpolation; K is the image degradation matrix, which is the cyclic Toeplitz matrix obtained by k; Ψ is a sparse base, which can be generated according to Fourier transform, discrete cosine transform, wavelet transform, or it can be an over-complete dictionary; $\tilde{x}$ is a sparse coefficient. The one-dimensional vectorized HR image $x=\Psi \tilde{x}$. The SR reconstruction can be completed by solving the following objective function:

(3) $${\tilde{x}}^{\prime }=\arg\underset{\tilde{x}}{\min\|}SK\Psi \tilde{x}-y{\|}_{2}^{2}+\omega \|\tilde{x}{\|}_{0}$$

where $\|\tilde{x}{\|}_{0}$ represents the number of non-zero elements in $\tilde{x}$. However, because Equation (3) is an NP-hard problem. Donoho [24] pointed out that the equivalent solution can be obtained by solving the L1 optimization problem. The objective function can be expressed as:

(4) $${\tilde{x}}^{\prime }=\arg\underset{\tilde{x}}{\min\|}SK\Psi \tilde{x}-y{\|}_{2}^{2}+\omega \|\tilde{x}{\|}_{1}$$

The most sparse $\tilde{x}$ is solved by the optimization problem of Equation (4), and the result of image SR reconstruction can be obtained by $x=\Psi \tilde{x}$. When K is also unknown, the optimization problem of Equation (4) is transformed into a blur kernel estimation problem:

(5) $$\left({\tilde{x}}^{\prime },K^{\prime }\right)=\arg\underset{\tilde{x},K}{\min\|}SK\Psi \tilde{x}-y{\|}_{2}^{2}+\omega \|\tilde{x}{\|}_{1}$$

Obviously, due to the undetermined nature of the problem, the estimated value of K cannot be obtained directly from Equation (5), so it is necessary to introduce prior information to constrain and optimize the choice of solutions. There are obvious differences in the color brightness of infrared images with different pseudo-color conversion methods. Therefore, we chose the image Extreme Channels Prior mentioned in [25] as a constraint and introduce it into Equation (5) to improve the accuracy of blur kernel estimation.

### 2.2. Priori of Extreme Value Channel of Infrared Image of Power Equipment

In this section, we introduce the image Extreme Channels Prior. There are many types of prior information that can be used to estimate the image blur kernel, such as L0-regularized gradient prior, dark channel prior and Extreme Channels Prior. The reasons why we chose Extreme Channels Prior are as follows: In the infrared image of power equipment, the faulty heating part and the edge texture part of the equipment contain the most important information. The faulty heating part is the focus of infrared diagnosis, and it often appears as the brightest part in the image. The edge texture part is the main basis for image segmentation and target recognition, which is often the darkest part. The Extreme Channels Prior used in this article is calculated based on the local brightest and darkest pixel values in the infrared image before and after blurring. Therefore, Extreme Channels Prior pays more attention to the processing of local brightness extremes in the image and can use this as the focus to estimate the blur kernel, thereby improving the visual quality of the faulty heating part and the edge texture part of the final reconstructed image. This feature can make the infrared image of the power equipment reconstructed by the method more suitable for the needs of practical applications.

**Figure 1:**
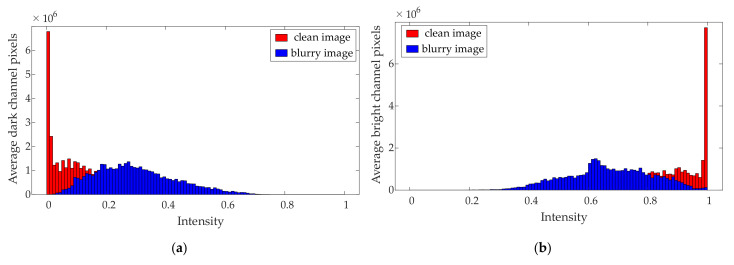
Extreme channels of infrared images before and after blurring: (**a**) dark channel of clear and blurred images; (**b**) bright channel of clear and blurred images.

It should be noted that this paper does not directly process the infrared image in RGB mode, but converts it into YCbCr mode. Y is the luminance component, Cb is the blue chrominance component, and Cr is the red chrominance component. Since the human eye is more sensitive to the Y component, the visual difference caused by the subtle changes of the other two components is extremely small, so the following image Extreme Channels Prior statistical analysis will take the Y component as an example. Specifically, Extreme Channels includes a bright channel component and a dark channel component, which are, respectively, arranged by the local maximum and minimum values in the Y component according to a certain rule. For image X, the components of the bright channel and dark channel are:

(6) $$B\left(X\right)\left(p\right)=\underset{q\in N\left(p\right)}{\max}\left(X\left(q\right)\right)$$

(7) $$D\left(X\right)\left(p\right)=\underset{q\in N\left(p\right)}{\min}\left(X\left(q\right)\right)$$

where p and q donate pixel locations; $N\left(p\right)$ is an image patch centered at p; $B\left(\cdot \right)$ and $D\left(\cdot \right)$ are functions for finding the local maximum and local minimum of the image, respectively. For a general image, after the pixel brightness value is normalized, most of the values obtained by $B\left(X\right)$ and $D\left(X\right)$ should be distributed at both ends of the interval $\left[0,1\right]$, respectively. However, the convolution operation of the blur kernel on the clear image will change the extreme value distribution of the image. Because the convolution operation is a weighted summation of the pixel values in the local neighborhood, it will generally cause the minimum value of the pixel value in the neighborhood to become larger and the maximum value to decrease. The mathematical basis has been proved in the literature [25,26]. Figure 1 is a statistical result of the difference between the local maximum and the local minimum distribution of 100 infrared images of power equipment under clear and blur conditions. It can be seen that the above rules are also applicable to infrared images of power equipment. Therefore, the introduction of Extreme Channels Prior can effectively distinguish between clear infrared images and blurred infrared images, prompting the intermediate latent image to move closer to the clear image, thereby ensuring the accuracy of the blur kernel estimation.

### 2.3. Blur Kernel Estimation Model

In this section, we will propose a blur kernel estimation model based on maximum a posterior probability (MAP) framework and the basic SR method of compressed sensing. The MAP framework is:

(8) $$\left(k^{\prime },X^{\prime }\right)=\arg\underset{k,X}{\min}X\left(k⊗X,Y\right)+\beta_{k}p\left(k\right)+\beta_{X}p\left(X\right)$$

where $p\left(k\right)$ and $p\left(X\right)$ are the priori information of k and X, respectively. According to the statistics and analysis in Section 2.2, we use the $L_{0}$ norm regular term based on Extreme Channels Prior as the prior information of the image to be reconstructed:

(9) $$p\left(X\right)=\|D\left(X\right){\|}_{0}+\|1-B\left(X\right){\|}_{0}$$

Combined with the SR model of compressed sensing, the objective function of blur kernel estimation can be constructed:

(10) $$\begin{array}{l} \left(k^{\prime },X^{\prime }\right)=\arg\underset{k,X}{\min}\|S_{l}\left(k⊗X\right)S_{r}-Y{\|}_{2}^{2}+\delta \|\nabla X{\|}_{0}+\eta \|k{\|}_{2}^{2}\\ \;\;\;\;\;\;\;\;\;\;\;\;\;\;+\gamma \|D\left(X\right){\|}_{0}+\mu \|1-B\left(X\right){\|}_{0}+\rho \|\Psi^{T}X{\|}_{1} \end{array}$$

where δ,η,γ,μ and ρ are weight coefficients. The first term of the equation is the data fidelity term, which is used to ensure that there is a corresponding relationship between X and Y and we use the $L_{2}$ norm to constrain the difference. The second term is used to preserve the significant gradient of the image and remove the small gradient, thereby improving the accuracy of the blur kernel estimation. The third term is the constraint on the sparseness of the blur kernel. The fourth item is used to preserve the sparse characteristic of the minimum value in the brightness component of the infrared image. The last item is used to ensure that the sparse coefficients obtained after the sparse transformation of the image are sparse enough and is combined with the first item to achieve the goal of SR. In order to ensure the calculation efficiency of the blur kernel estimation, we did not stretch the image into a column vector in the original compressed sensing before the calculation. The reason for this is that if the image is not divided into blocks but directly stretched as a column vector for calculation, the downsampling matrix will be too large and the calculation speed will be greatly reduced. Therefore, we modified the original model slightly, constructing a row sampling matrix $S_{l}$ and a column sampling matrix $S_{r}$ according to the principle of cubic interpolation so that the downsampling operation is performed twice. The size of the downsampling matrix and the position of non-zero elements are determined according to the downsampling rate, and the element values are determined according to the cubic interpolation downsampling function. The method of solving Equation (10) is introduced in Section 4.

## 3. Non-Blind SR Reconstruction Model

### 3.1. Non-Blind SR Objective Function

After completing the estimation of k, the basic non-blind SR model is:

(11) $$X^{\prime }=\arg\underset{X}{\min}\|S_{l}\left(k⊗X\right)S_{r}-Y{\|}_{2}^{2}+\rho \|\Psi^{T}X{\|}_{1}$$

Equation (11) does not contain any prior information except for the sparse constraint. It is difficult to achieve the effect of deconvolution and deblurring due to the ill-posedness of the problem using the image reconstructed directly from Equation (11). Therefore, it is necessary to introduce other prior information as constraints according to the law of image statistics.

**Figure 2:**
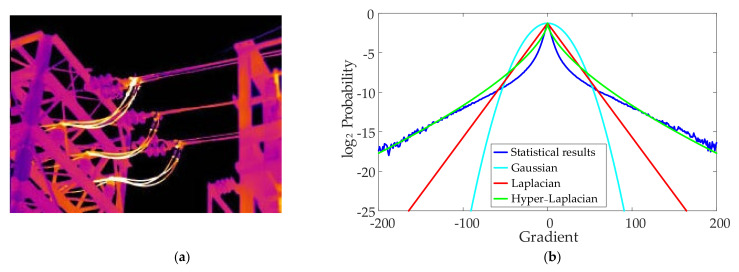
Fitting results of infrared image gradient probability density curve and different distributions: (**a**) typical infrared image; (**b**) fitting effects of different distributions.

Currently, the best deblurring method generally uses a set of filter output edge statistics to match the edge statistics of the clear image as a priori constraint for the deblurring problem. The Gaussian distribution, Laplacian distribution and Hyper-Laplacian distribution are commonly used to fit the image gradient. Assuming that the edge distribution is Gaussian, the deblurring problem has an analytical solution in the frequency domain, and the image can be restored efficiently through fast Fourier transform. However, a clear infrared image usually has a non-Gaussian edge, as shown in Figure 2, so the Gaussian distribution fitting the image gradient will lead to poor visual effects of the reconstruction result. Another common method is to assume that the edges of the image conform to the Laplacian distribution. However, due to the “heavy tail” characteristic of the edge distribution of the image, the effect of Laplace distribution fitting is also not good. Therefore, most of the methods at this stage adopt the Hyper-Laplacian distribution that can better fit the “heavy tail” characteristic of the image edge as the prior information, and then achieve the purpose of deconvolution and deblurring. Figure 2 shows the probability density curve obtained by statistically calculating the gradient of the infrared image and the fitting effect of different distributions.

Therefore, it can be seen from the above discussion that a prior constraint of image edge distribution needs to be introduced for Equation (11). No matter what kind of distribution is used to fit the image edge, it can be written as $p\left(X\right)\propto e^{-\lambda {\left|X\right|}^{\alpha }}$, where 0<α≤2. If 0<α<1, it is Hyper-Laplacian distribution, α=1 is Laplace distribution, and α=2 is Gaussian distribution. According to Bayes’ theorem, the maximum posterior probability solution of X is:

(12) $$X^{\prime }=\arg\underset{X}{\min}X\left(k⊗X,Y\right)+\beta_{X}p\left(X\right)$$

where $p\left(X\right)=\sum_{i}\lambda \|f_{i}⊗X{\|}_{\alpha }^{\alpha }$. λ is the strength coefficient of the prior constraint of the edge distribution. $f_{i}$ is the derivative filter of each order, $i=\left\{x,y,xx,yy,xy\right\}$. The non-blind SR objective function becomes:

(13) $$X^{\prime }=\arg\underset{X}{\min}\|S_{l}\left(k⊗X\right)S_{r}-Y{\|}_{2}^{2}+\rho \|\Psi^{T}X{\|}_{1}+\underset{i}{\sum }\lambda \|f_{i}⊗X{\|}_{\alpha }^{\alpha }$$

### 3.2. Adaptive Regularization Intensity Adjustment Method

**Figure 3:**
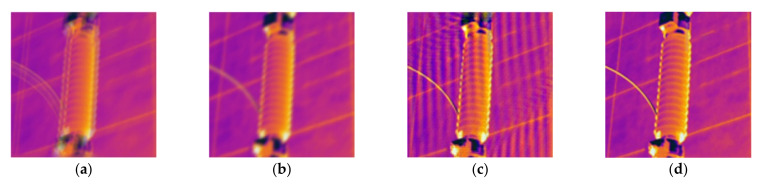
Infrared image reconstruction results under different regularization intensity: (**a**) LR image; (**b**) strongly regularized reconstruction results; (**c**) weak regularization reconstruction results; (**d**) results of the proposed method.

The non-blind super-resolution objective function is determined in Section 3.1, as shown in Equation (13). However, the model still needs to be further improved. The reason is that the value of λ needs to consider the difference in the semantics of the pixels within the image. The same value should not be used to constrain the entire picture. In this way, the edge texture of the reconstructed image is blurred when the regularization is strong, and the reconstructed image appears artifact ringing when the regularization is weak. Figure 3 shows the reconstruction results of infrared images processed with different regularization intensities and our proposed adaptive method.

It can be seen from Figure 3c that when strong regularization is used to constrain the prior information of the image edge, that is, when $\lambda =0.1\times 10^{-1}$, the reconstruction result does not contain ringing artifacts. However, the edge texture of the device is blurred, the image contrast is low, and the visual effect is poor. As shown in Figure 3d, when weak regularization is used for constraints, that is, when $\lambda =0.1\times 10^{-4}$, the reconstructed image is clearer and the edge texture contrast of the device is high, but the smooth area in the image has an obvious ringing effect. However, we extract the significant edge regions in the reconstructed image by using a double-prior quadratic estimation method, distinguish the edge regions and smooth regions according to the generated label images, and adjust the intensity of the regular term with different λ values. Therefore, the reconstruction effect is better. As shown in Figure 3b, the edge texture of the reconstructed image is clear and does not contain ringing.

**Figure 4:**
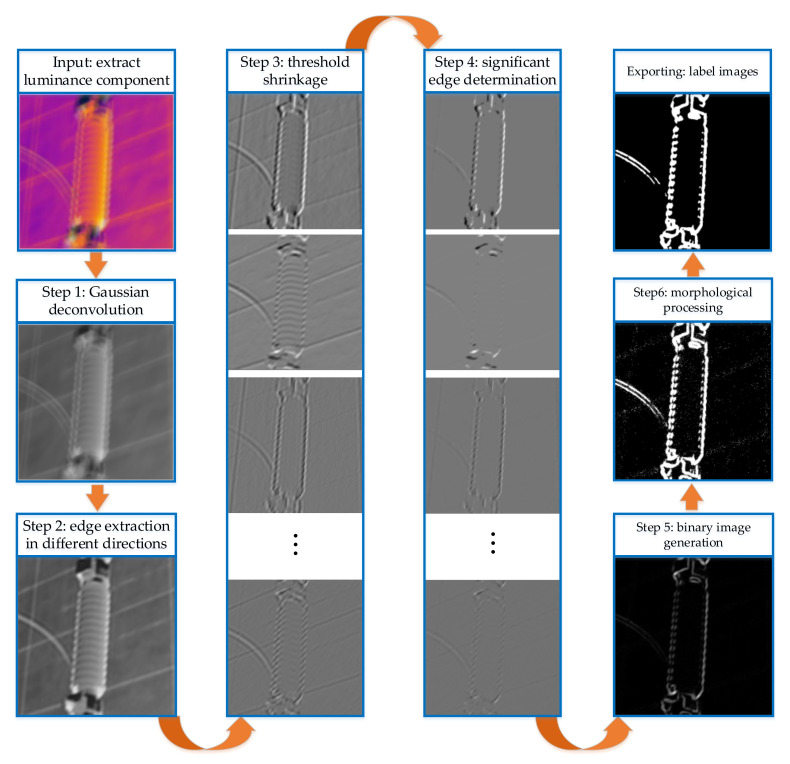
Flow chart of adaptive regularization intensity adjustment method.

Figure 3 shows the necessity of adaptive control of the regularization intensity in the reconstruction process. The following is an introduction to the regular term intensity adjustment method of the double-prior quadratic estimation that we adopted, and the flowchart is shown in Figure 4.

The adaptive regular term intensity adjustment method we adopt is to use different priors as constraints to reconstruct the image twice according to the model of Equation (13). In the first reconstruction, the model uses a Gaussian prior which is easy to solve, extracts the significant edges of the reconstructed image, and generates the label image. The secondary reconstruction adopts the Hyper-Laplacian prior as the constraint, and adaptively adjusts the regularization intensity of different pixels according to the label image. The specific steps in Figure 4 are described as follows:

Step 1: Let α=2 in Equation (13), and solve it to obtain the preliminary reconstructed image $X_{1}$.

Step 2: Use filter bank $\left\{f_{x},f_{y},f_{xx},f_{yy},f_{xy}\right\}$ to filter $X_{1}$ to obtain edge images in all directions.

Step 3: Perform threshold shrinkage on edge images; the shrinking method is:

(14) $$X_{i}=\frac{f_{i}⊗X_{1}}{{\left(\frac{\tau_{i}}{f_{i}⊗X_{1}}\right)}^{4}+1}$$

where $\tau_{i}=\sigma \times \max\left(\left|f_{i}⊗X_{1}\right|\right)$ is the shrinkage threshold; σ is the proportional coefficient; the shrinkage result is denoted as $X_{i}=\left\{X_{x},X_{y},X_{xx},X_{yy},X_{xy}\right\}$.

Step 4: Solve $\nabla X=\sum_{i}X_{i}$, and integrate the significant edges in all directions into the final image significant edge result ∇X.

Step 5: Set the elements smaller than $\sum_{i}\tau_{i}/10$ in ∇X to 0, and set the remaining elements to 1, thereby generating a binary image.

Step 6: Perform mathematical morphological processing on the binarized image. The opening and closing operations are performed once to remove the binarized image noise, and the final label image $X_{lab}$ is obtained.

After $X_{lab}$ is obtained, the value of λ can be adaptively controlled by:

(15) $$\lambda_{\left(m,n\right)}=\left\{\begin{array}{cc} 0.5\times 10^{-2}, & X_{lab\left(m,n\right)}=0\\ 2.5\times 10^{-4}, & X_{lab\left(m,n\right)}=1 \end{array}\right.$$

Let α=2/3 in Equation (13), and substitute $\lambda_{\left(m,n\right)}$ into Equation (13) to complete image SR reconstruction. In addition, the method of solving Equation (13) under different values of α is introduced in Section 4.

## 4. Model Solution

In Section 2 and Section 3, we, respectively, established the blur kernel estimation model and the non-blind SR reconstruction model as shown in Equations (10) and (13). This section will introduce their solution methods. In order to facilitate the solution, we use the semi-quadratic split method to introduce auxiliary variables for them, and then use the alternate minimization method to solve the unknown variables in the model. After introducing auxiliary variables, the blur kernel estimation model becomes:

(16) $$\begin{array}{l} \left(k^{\prime },X^{\prime },G^{\prime },a^{\prime },b^{\prime },c^{\prime },{\tilde{X}}^{\prime }\right)=\arg\underset{k,X,G,a,b,c,\tilde{X}}{\min}\|S_{l}GS_{r}-Y{\|}_{2}^{2}+\varepsilon \|k⊗X-G{\|}_{2}^{2}\\ +\delta \|a{\|}_{0}+\delta^{\prime }\|a-\nabla X{\|}_{2}^{2}+\eta \|k{\|}_{2}^{2}+\gamma \|b{\|}_{0}+\mu \|c{\|}_{0}+\rho \|\tilde{X}{\|}_{1}\\ +\gamma^{\prime }\|D\left(X\right)-b{\|}_{2}^{2}+\mu^{\prime }\|1-B\left(X\right)-c{\|}_{2}^{2}+\rho^{\prime }\|\Psi^{T}X-\tilde{X}{\|}_{2}^{2} \end{array}$$

where $\varepsilon ,\delta^{\prime },\gamma^{\prime },\mu^{\prime }$ and $\rho^{\prime }$ are penalty parameters. $a=\left\{a_{h},a_{v}\right\}$, $\nabla X=\left\{\nabla_{h}X,\nabla_{v}X\right\}$, $\nabla_{h}=\left[1,-1\right],\nabla_{v}={\left[1,-1\right]}^{T}$ are the row and column difference operators, respectively. After the introduction of auxiliary variables, Equation (13) becomes:

(17) $$\begin{array}{ll} \left(X^{\prime },G^{\prime },{\tilde{X}}^{\prime }\right) & =\arg\underset{X,G,\tilde{X}}{\min}\|S_{l}GS_{r}-Y{\|}_{2}^{2}+\varepsilon \|k⊗X-G{\|}_{2}^{2}+\underset{i}{\sum }\lambda \|f_{i}⊗X{\|}_{\alpha }^{\alpha }\\ & +\rho \|\tilde{X}{\|}_{1}+\rho^{\prime }\|\Psi^{T}X-\tilde{X}{\|}_{2}^{2} \end{array}$$

Equations (16) and (17) both contain variables G and $\tilde{X}$, and we solve them by the same objective function, so they are solved in the same way. We solve for G by:

(18) $$G^{\prime }=\arg\underset{G}{\min}\|S_{l}GS_{r}-Y{\|}_{2}^{2}+\varepsilon \|k⊗X-G{\|}_{2}^{2}$$

Equation (18) is a typical least squares problem, which can be solved by the gradient descent method. The derivative of Equation (18) with respect to G:

(19) $$d_{G}=2S_{l}^{T}\left(S_{l}GS_{r}\right)S_{r}^{T}-2\varepsilon \left(k⊗X-G\right)$$

The number of iterations and step length are determined by the one-step steepest descent scheme introduced in [27]. We solve for $\tilde{X}$ by:

(20) $${\tilde{X}}^{\prime }=\arg\underset{\tilde{X}}{\min}\rho \|\tilde{X}{\|}_{1}+\rho^{\prime }\|\Psi^{T}X-\tilde{X}{\|}_{2}^{2}$$

which can be solved by shrinking the soft threshold:

(21) $$\tilde{X}=\max\left\{\left|\Psi^{T}X\right|-\frac{\rho }{2\rho^{\prime }},0\right\}\circ \mathrm{sign}\left(\Psi^{T}X\right)$$

In addition to the common variables, Equations (16) and (17) also contain their own unique variables, and their solutions are introduced separately below. The variables a,b and c in Equation (16) are all constrained by the $L_{0}$ norm, which can be solved by hard threshold shrinkage. We solve for them by:

(22) $$a^{\prime }=\arg\underset{a}{\min}\delta \|a{\|}_{0}+\delta^{\prime }\|a-\nabla X{\|}_{2}^{2}$$

(23) $$b^{\prime }=\arg\underset{b}{\min}\gamma \|b{\|}_{0}+\gamma^{\prime }\|D\left(X\right)-b{\|}_{2}^{2}$$

(24) $$c^{\prime }=\arg\underset{c}{\min}\mu \|c{\|}_{0}+\mu^{\prime }\|1-B\left(X\right)-c{\|}_{2}^{2}$$

Their solutions are:

(25) $$\left(a_{h},a_{v}\right)=\left\{\begin{array}{cc} \left\{\nabla_{h}X,\nabla_{v}X\right\}, & {\left(\nabla_{h}X\right)}^{2}+{\left(\nabla_{v}X\right)}^{2}\geq \delta /\delta^{\prime }\\ 0, & \mathrm{otherwise} \end{array}\right.$$

(26) $$b=\left\{\begin{array}{cc} D\left(X\right), & {\left(D\left(X\right)\right)}^{2}\geq \gamma /\gamma^{\prime }\\ 0, & \mathrm{otherwise} \end{array}\right.$$

(27) $$c=\left\{\begin{array}{cc} 1-B\left(X\right), & {\left(1-B\left(X\right)\right)}^{2}\geq \mu /\mu^{\prime }\\ 0, & \mathrm{otherwise} \end{array}\right.$$

The variables X and k in Equation (16) are both constrained by the $L_{2}$ norm, which can be solved by the method of fast Fourier transform. We solve for X by:

(28) $$\begin{array}{l} X^{\prime }=\arg\underset{X}{\min}\varepsilon \|k⊗X-G{\|}_{2}^{2}+\delta^{\prime }\|a-\nabla X{\|}_{2}^{2}+\rho^{\prime }\|\Psi^{T}X-\tilde{X}{\|}_{2}^{2}\\ \;\;\;+\gamma^{\prime }\|D\left(X\right)-b{\|}_{2}^{2}+\mu^{\prime }\|1-B\left(X\right)-c{\|}_{2}^{2} \end{array}$$

In order to maintain the consistency between $D\left(X\right)$ and $1-B\left(X\right)$ to facilitate the solution, by the operational nature of the $B\left(X\right)$ and $D\left(X\right)$ functions, $1-B\left(X\right)$ can be equivalent to $D\left(1-X\right)$. In addition, due to the non-linearity of the function $D\left(X\right)$, the equivalent linear operator M is introduced for it. M is essentially a mapping matrix, and its construction method is:

(29) $$M\left(p,q\right)=\left\{\begin{array}{cc} 1, & q=\arg\underset{q\in N\left(p\right)}{\min}X\left(q\right)\\ 0, & \mathrm{otherwise} \end{array}\right.$$

The function of the M matrix is to transfer the minimum value in the image block centered on the p pixel (i.e., the value of the q pixel) to the p pixel. As the transposed matrix of M, $M^{T}$ plays a role of reverse rearrangement during operation. Reverse rearrangement means that the pixel value at position p is used to reversely replace the pixel value at position q. Therefore, Equation (28) can be expressed as:

(30) $$\begin{array}{l} X^{\prime }=\arg\underset{X}{\min}\varepsilon \|k⊗X-G{\|}_{2}^{2}+\delta^{\prime }\|a-\nabla X{\|}_{2}^{2}+\rho^{\prime }\|\Psi^{T}X-\tilde{X}{\|}_{2}^{2}\\ \;\;\;+\gamma^{\prime }\|M_{X}X-b{\|}_{2}^{2}+\mu^{\prime }\|M_{1-X}\left(1-X\right)-c{\|}_{2}^{2} \end{array}$$

The solution of (30) can be obtained by FFT:

(31) $$X=F^{-1}\left(\frac{\varepsilon \bar{F\left(k\right)}\circ F\left(G\right)+\delta^{\prime }F_{a}+\gamma^{\prime }F\left(M_{X}^{T}b\right)+\mu^{\prime }F\left(M_{1-X}^{T}c-1\right)+\rho^{\prime }F\left(\Psi^{T}\tilde{X}\right)}{\varepsilon \bar{F\left(k\right)}\circ F\left(k\right)+\delta^{\prime }F_{\nabla }+\gamma^{\prime }+\mu^{\prime }+\rho^{\prime }}\right)$$

where $F_{a}$ is $\bar{F\left(\nabla_{h}\right)}\circ F\left(a_{h}\right)+\bar{F\left(\nabla_{v}\right)}\circ F\left(a_{v}\right)$; $F_{\nabla }$ is $\bar{F\left(\nabla_{h}\right)}\circ F\left(\nabla_{h}\right)+\bar{F\left(\nabla_{v}\right)}\circ F\left(\nabla_{v}\right)$; $F\left(\cdot \right)$ and $F^{-1}\left(\cdot \right)$ denote the fast Fourier transform and inverse fast Fourier transform, respectively; $\bar{F\left(\cdot \right)}$ is the complex conjugate operator; ∘ denotes component multiplication, and the division in Formula (31) is component division. It should be noted that M and $M^{T}$, as linear operators, did not actually generate a matrix and perform matrix multiplication during the calculation process, but instead set up a lookup table according to its meaning. For example, $M_{X}^{T}b$ does not actually calculate the product of the matrix $M_{X}^{T}$ and b. Instead, according to the relationship that b is approximately equal to $M_{X}X$, the minimum value element in X is replaced with the element in b to obtain the result of $M_{X}^{T}b$. This avoids the generation and calculation of large matrices in the algorithm, and significantly improves the running speed.

We estimate the blur kernel k by:

(32) $$k^{\prime }=\arg\underset{k}{\min}\eta \|k{\|}_{2}^{2}+\varepsilon \|k⊗X-G{\|}_{2}^{2}$$

For the subproblem k, directly using the intermediate latent image to estimate the blur kernel is not accurate [28]; therefore, the gradient image is used to estimate the blur kernel. Then, the solution of k can be obtained by solving the following:

(33) $$k^{\prime }=\arg\underset{k}{\min}\eta \|k{\|}_{2}^{2}+\varepsilon \|k⊗\nabla X-\nabla G{\|}_{2}^{2}$$

The solution of (33) can be obtained by FFT:

(34) $$k=F^{-1}\left(\frac{\varepsilon \bar{F\left(\nabla X\right)}F\left(\nabla G\right)}{\varepsilon \bar{F\left(\nabla X\right)}F\left(\nabla X\right)+\eta }\right)$$

Since the blur kernel k≥0 and $\|k{\|}_{1}=1$, after each iteration of the k subproblem, we set the negative elements of k to zero and normalize k at the end. The solution of all variables in the process of blur kernel estimation has been given, and Algorithm 1 shows the main steps for the blur kernel estimation algorithm. As suggested by [25,26,29], we decrease μ,γ,δ gradually to make more information available for kernel estimation.
**Algorithm 1:** Blur Kernel Estimation AlgorithmInput: Blurred image Y generate the initial value of each variable for i=1:5 do $\varepsilon \leftarrow \varepsilon_{0}$
 repeat
 solve for G using the gradient descent method, $\gamma^{\prime }\leftarrow 2\gamma ,\mu^{\prime }\leftarrow 2\mu$. repeat solve for b using (26), solve for c using (27), $\rho^{\prime }\leftarrow 2\rho$. repeat solve for $\tilde{X}$ using (21), $\delta^{\prime }\leftarrow 2\delta$. repeat solve for a using (25),solve for X using (31), $\delta^{\prime }\leftarrow 2\delta$. until $\delta^{\prime }>\delta_{max}^{\prime }$ $\rho^{\prime }\leftarrow 2\rho^{\prime }$. until $\rho^{\prime }>\rho_{max}^{\prime }$
$\gamma^{\prime }\leftarrow 2\gamma^{\prime },\mu^{\prime }\leftarrow 2\mu^{\prime }$.
$\mathrm{until}\gamma^{\prime }>\gamma_{max}^{\prime }$ and $\mu^{\prime }>\mu_{max}^{\prime }$ ε←4ε.
 until $\varepsilon >\varepsilon_{max}$ solve for k using (34).  μ←0.9μ,γ←0.9γ,δ←0.9δ. end for 
Output: blur kernel k.


Finally, only the solution of X in Equation (17) has not been given yet. When the value of α is different, the solution method is different. When α=2, we solve for X by:

(35) $$X^{\prime }=\arg\underset{X}{\min}\varepsilon \|k⊗X-G{\|}_{2}^{2}+\rho^{\prime }\|\Psi^{T}X-\tilde{X}{\|}_{2}^{2}+\underset{i}{\sum }\lambda \|f_{i}⊗X{\|}_{2}^{2}$$

The purpose of initial reconstruction using Gaussian prior is only to extract the significant edges of the image. Therefore, λ should be a larger value to suppress the generation of ringing artifacts,$\;\lambda =0.1\times 10^{-2}$. The solution of (35) can be obtained by FFT:

(36) $$X=F^{-1}\left(\frac{\varepsilon \bar{F\left(k\right)}\circ F\left(G\right)+\rho^{\prime }F\left(\Psi^{T}\tilde{X}\right)}{\varepsilon \bar{F\left(k\right)}\circ F\left(k\right)+\rho^{\prime }+\lambda \sum_{i}\bar{F\left(f_{i}\right)}\circ F\left(f_{i}\right)}\right)$$

When α=2/3, the non-blind SR model is constructed by adaptive regularization, and we solve for X by:

(37) $$\begin{array}{l} X^{\prime }=\arg\underset{X}{\min}\varepsilon \|k⊗X-G{\|}_{2}^{2}+\rho^{\prime }\|\Psi^{T}X-\tilde{X}{\|}_{2}^{2}\\ \;\;\;\;\;+\overset{M}{\underset{m}{\sum }}\overset{N}{\underset{n}{\sum }}\left(\underset{i}{\sum }\lambda_{\left(m,n\right)}{\left|{\left(f_{i}⊗X\right)}_{\left(m,n\right)}\right|}^{\frac{2}{3}}\right) \end{array}$$

where M and N are the number of pixels in the row and column direction of the image, respectively; $\lambda_{\left(m,n\right)}$ can be obtained according to Equation (15). In order to facilitate the solution, the auxiliary variable $w_{i}$ is introduced by the semi-quadratic split method. Equation (37) can be expressed as:

(38) $$\begin{array}{c} \left(X^{\prime },w_{i}^{\prime }\right)=\arg\underset{X,w_{i}}{\min}\varepsilon \|k⊗X-G{\|}_{2}^{2}+\rho^{\prime }\|\Psi^{T}X-\tilde{X}{\|}_{2}^{2}\\ +\underset{i}{\sum }\left(\overset{M}{\underset{m}{\sum }}\overset{N}{\underset{n}{\sum }}\lambda_{\left(m,n\right)}{\left|{\left(w_{i}\right)}_{\left(m,n\right)}\right|}^{\frac{2}{3}}+\vartheta \|f_{i}⊗X-w_{i}{\|}_{2}^{2}\right) \end{array}$$

Equation (38) can be divided into the X sub-problem and the w sub-problem to be solved separately. We solve the X sub-problem by:

(39) $$X^{\prime }=\arg\underset{X}{\min}\varepsilon \|k⊗X-G{\|}_{2}^{2}+\rho^{\prime }\|\Psi^{T}X-\tilde{X}{\|}_{2}^{2}+\underset{i}{\sum }\xi \|f_{i}⊗X-w_{i}{\|}_{2}^{2}$$

The solution is the same as (35). X can be obtained by FFT:

(40) $$X=F^{-1}\left(\frac{\varepsilon \bar{F\left(k\right)}\circ F\left(G\right)+\rho^{\prime }F\left(\Psi^{T}\tilde{X}\right)+\xi \sum_{i}\bar{F\left(f_{i}\right)}\circ F\left(w_{i}\right)}{\varepsilon \bar{F\left(k\right)}\circ F\left(k\right)+\rho^{\prime }+\xi \sum_{i}\bar{F\left(f_{i}\right)}\circ F\left(f_{i}\right)}\right)$$

After obtaining X, we solve the w sub-problem. Let $f_{i}⊗X=v$, then the objective function can be abbreviated as:

(41) $$w^{\prime }=\arg\underset{X,w_{i}}{\min}\lambda {\left|w\right|}^{\frac{2}{3}}+\xi {\left(v-w\right)}^{2}$$

We set the derivative of Equation (41) to be 0:

(42) $$\frac{2\lambda }{3}{\left|w\right|}^{-\frac{1}{3}}sign\left(w\right)+2\xi \left(w-v\right)=0$$

We further transform Equation (42) into:

(43) $$w^{4}-3vw^{3}+3v^{2}w^{2}-v^{3}w+\frac{\lambda^{3}}{27\xi^{3}}=0$$

The root of Equation (43) is r. According to [30], when r is between v/2 and v, the solution of Equation (43) is $w^{\prime }=r$, otherwise $w^{\prime }=0$. However, since the solution of Equation (43) only depends on the ratio of λ and ξ and the value of the variable v, it can be solved by a lookup table (LUT). Where ξ is an integer power of $\sqrt{2}$ between 1 and 256, λ is $0.5\times 10^{-2}$ or $2.5\times 10^{-4}$, and v is 15,000 different values between −0.9 and 0.9. Solving Equation (43) in turn can form an offline lookup table. The LUT can give a solution to the objective function with an accuracy close to that of the analytical method at a faster speed [30].

**Figure 5:**
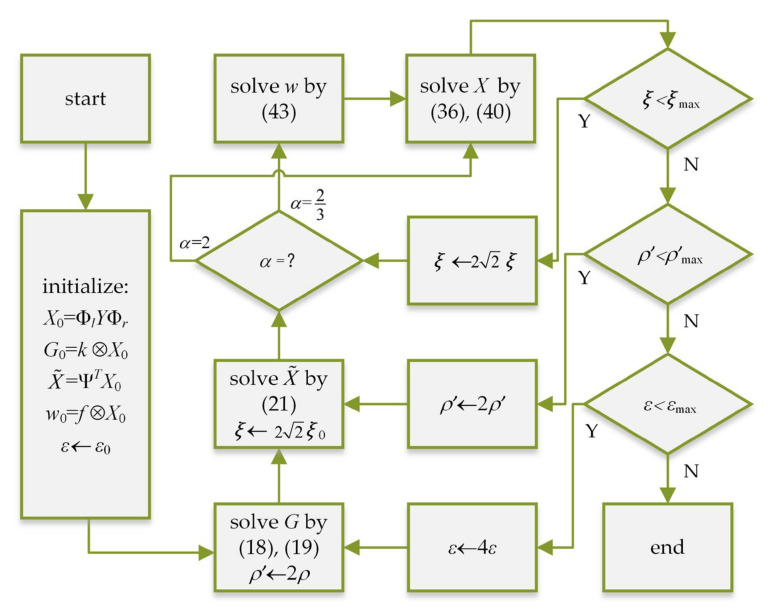
Non-blind SR flow chart.

We, respectively, give the solution of each unknown variable in the objective function of blur kernel estimation and non-blind SR. When α takes different values, the non-blind SR reconstruction process is shown in Figure 5.

## 5. Experiment and Result Analysis

Our test environment parameters were as follows: Intel(R) Core(TM)i5-9300H CPU @2.40 GHz; memory: 16.00 GB; operating system: Windows 10; MATLAB R2019a. We obtained the following fixed parameters through repeated experiments and adjustments: δ=γ=μ=0.004; $\varepsilon_{0}=0.25$; η=ρ=ξ=1; σ=0.3. The image block size used for the dark channel search was 35 × 35. The sparse base Ψ used the Daubechies 8 wavelet base.

In order to make the experimental results more convincing, in addition to comparing our method with the classical blind SR method, we also design comparative experiments for the blur kernel estimation and non-blind SR reconstruction in our method. In the blind SR comparison experiment, we compared our method with the methods proposed by Keys [31], Shao [20], Michaeli [23], and Kim [22]. Since the actual infrared image of the power equipment did not have the original, clear HR image, we adopted two other objective evaluation indicators: average gradient (AG) and information entropy (IE). The calculation method of AG is as follows:

(44) $$AG=\frac{1}{MN}\underset{i}{\sum }\underset{j}{\sum }\sqrt{f_{x}^{2}\left(i,j\right)+f_{y}^{2}\left(i,j\right)}$$

where $f_{x}\left(i,j\right)$ and $f_{y}\left(i,j\right)$ are the image convolution results of the difference operator in the row and column directions, respectively. The larger the AG value is, the more drastically the grayscale changes in the image and the more the image levels, that is, the clearer the image is.

Entropy represents the uniformity of a system in physics. The more uniformly a system is distributed, the greater its information entropy is. The concept of image information entropy is derived from this, which can be defined as follows:

(45) $$IE=-\overset{n}{\underset{i=0}{\sum }}p\left(i\right)\log_{2}p\left(i\right)$$

where $p\left(i\right)$ represents the frequency of the pixel point with the gray value of i in the image. The larger the IE value, the richer the information contained in the image.

In addition, in order to prove the effectiveness of the blur kernel estimation method in this paper, we compared the blur kernels estimated by our method and the algorithms proposed in [20,23,32]. We used the sum of the squared differences error (SSDE) to evaluate the accuracy of the estimated blur kernel:

(46) $$SSDE=\underset{i}{\sum }\underset{j}{\sum }{\left(k_{est}\left(i,j\right)-k_{gt}\left(i,j\right)\right)}^{2}$$

where $k_{est}$ represents the estimated blur kernel and $k_{gt}$ represents the true blur kernel of the image.

Finally, in order to verify the performance of the non-blind SR reconstruction method in this paper, we use the known blur kernel to process the HR and clear infrared image according to Equation (1). Taking the synthetic infrared image and the known blur kernel as input, we compare our method with the existing non-blind SR methods. We select the methods proposed by Keys [31], Glasner [13], Dong [15] and Zhao [17] as the comparison method. Since the artificially synthesized infrared image had the original, clear HR image, the PSNR and SSIM evaluation indicators could be used to evaluate the reconstruction results.

### 5.1. Blind SR Comparison Experiment

**Figure 6:**
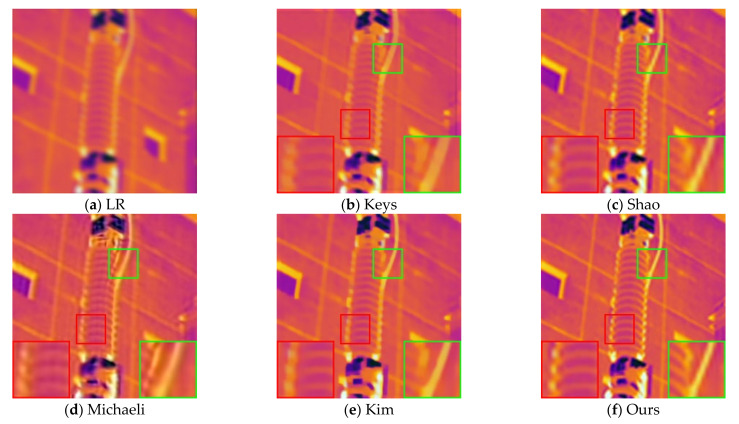
Reconstruction results of different methods: (**a**) LR infrared image; (**b**) reconstruction results using the method in [31] (AG = 32.169; IE = 5.932); (**c**) reconstruction results using the method in [20] (AG = 35.921; IE = 5.991); (**d**) reconstruction results using the method in [23] (AG = 40.862; IE = 6.073); (**e**) reconstruction results using the method in [22] (AG = 38.910; IE = 6.040); (**f**) reconstruction results using our method (AG = 43.280; IE = 6.161).

First, we reconstruct the LR infrared images of the power equipment that are actually collected to verify the effectiveness of the blind SR method in the practical application. For the experiment, we used eleven infrared images taken on site with a resolution of 128 × 128 for SR reconstruction. Figure 6 shows the images reconstructed using different methods for the 11th LR infrared image.

**Figure 7:**
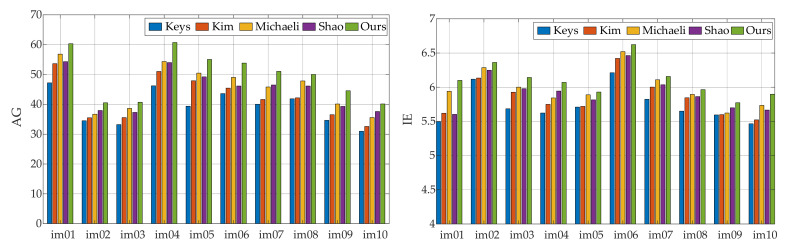
AG and IE parameter values of reconstruction results of different methods.

As shown in Figure 6, the method proposed by Key does not consider the influence of the blur kernel when reconstructing the image, and there is an inherent smoothness benefit of interpolation algorithms. The reconstruction result obtained by this method has the worst visual effect, and there is no obvious difference from the LR image. Compared with the original low-resolution image, the visual quality of the reconstructed image by Shao’s method has been significantly improved, but the detailed texture is still not clear enough. Obvious artifacts and ringing appear in the reconstruction results of Michaeli’s method. This is a common problem caused by improper regularization intensity in the SR reconstruction process, and it is also a problem that this paper focuses on improving and solving. Although the image reconstructed by Kim has higher contrast and brighter colors, according to the enlarged part in the green box, the edges are too smooth. On the whole, the edge texture of the image reconstructed by our method is the clearest, and there are no artifacts and ringing. This shows that our method has certain performance advantages compared with the comparison method. The AG and IE values of the remaining 10 images reconstructed using different methods are given in Figure 7. It can be seen that Kim and Shao’s methods are similar in performance. Michaeli’s method has a significantly higher reconstruction image index, which is due to improper control of the regularization intensity. Generally speaking, the infrared image reconstructed by our method has obvious advantages compared with the comparison methods in the evaluation index.

### 5.2. Experiment of Blur Kernel Estimation

**Figure 8:**
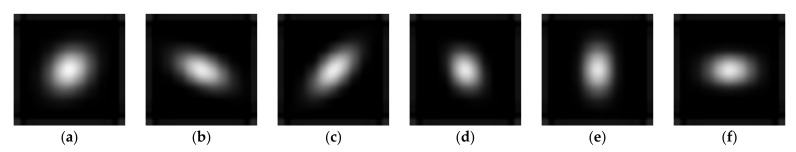
Six different blur kernels: (**a**) BK1; (**b**) BK2; (**c**) BK3; (**d**) BK4; (**e**) BK5; (**f**) BK6.

In order to prove the effectiveness of the blur kernel estimation method in this paper, we used the six blur kernels shown in Figure 8 to sequentially blur 100 infrared images and perform double downsampling. We used our method and the comparison methods to estimate the blur kernel based on the LR blurred image. For each blur kernel, the average SSDE parameters of the blur kernel estimated by the different methods on 100 synthetic blurred infrared images are shown in Table 1. It can be seen from the data in Table 1 that compared with the comparison methods, our blur kernel estimation method has achieved better results in accuracy.

### 5.3. Non-Blind SR Comparison Experiment

**Figure 9:**
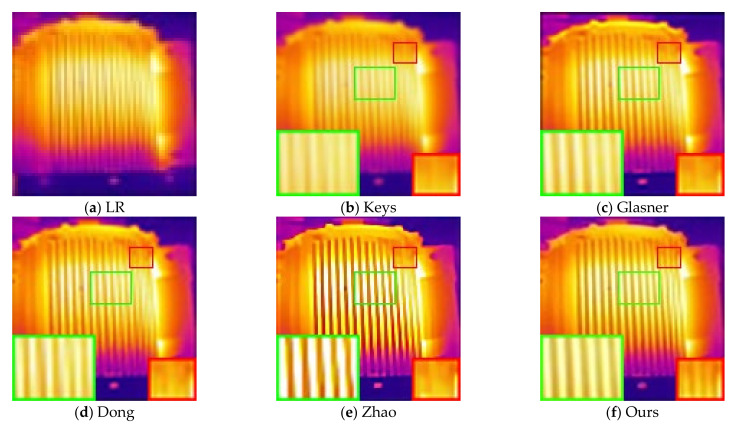
Reconstruction results of different methods when using BK6: (**a**) HR infrared image; (**b**) reconstruction results using the method in [31] (PSNR = 28.338 dB; SSIM = 0.911); (**c**) reconstruction results using the method in [13] (PSNR = 30.542 dB; SSIM = 0.927); (**d**) reconstruction results using the method in [15] (PSNR = 31.213 dB; SSIM = 0.952); (**e**) reconstruction results using the method in [17] (PSNR = 29.372 dB; SSIM = 0.940); (**f**) reconstruction results using our method (PSNR = 32.503 dB; SSIM = 0.963).

In this section, we use the six blur kernels in Section 5.2 to process 10 HR and clear infrared images according to Equation (1) to obtain 60 artificially synthesized LR images. Figure 9 shows the LR infrared image of the 10th HR image synthesized by BK6, as well as the reconstruction results of different non-blind SR methods.

**Figure 10:**
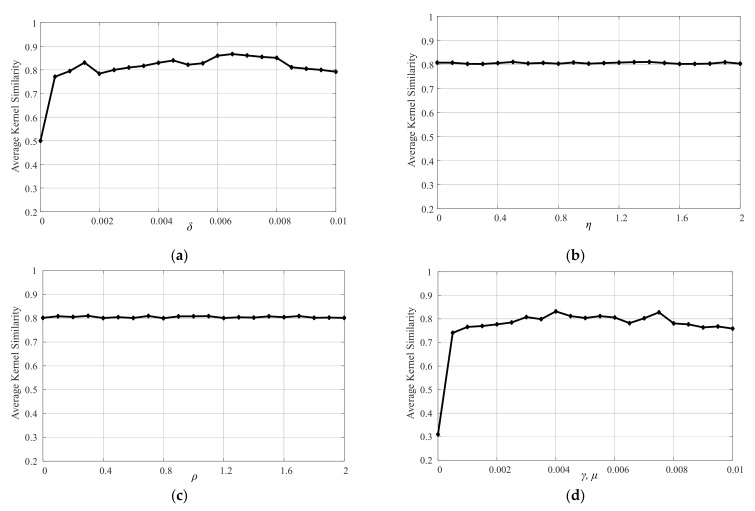
Sensitivity analysis of δ,η,γ,μ  and ρ for the blur kernel estimation model: (**a**) δ parameter; (**b**) η parameter; (**c**) ρ parameter; (**d**) γ and μ parameters.

It can be seen from Figure 9 that the method proposed by Keys has the worst visual effect due to the inherent smoothing benefits of interpolation algorithms, and the transformer texture is almost invisible. The visual quality of Glasner and Dong’s methods are similar, but the texture is not as clear as our proposed method, especially in small local details. The reconstruction result of Zhao’s method is somewhat distorted, and the image is too sharp, resulting in too high brightness. This will have a very bad influence in infrared diagnosis, and it is easy to cause the operation and maintenance personnel to misjudge the operating temperature of the equipment. Additionally, according to the part marked in the red box, its ability to reconstruct small textures obviously has a certain gap compared with our method. Due to the large number of images used in the experiment, the reconstruction results of the remaining images are given in the form of evaluation parameters. The PSNR and SSIM values of the reconstructed image of different algorithms are shown in Figure 10. Due to the large amount of data, for the reconstruction results of the same HR image processed by different blur kernels, the objective evaluation parameters are averaged and displayed. It can be seen from Table 2 and Table 3 that the performance of our method is significantly improved compared to the comparison methods.

### 5.4. Sensitivity Analysis

In this paper, the blur kernel estimation model involves many parameters. In this section, we analyze the influence of its value. The blur kernel estimation model involves five main parameters δ,η,γ,μ and ρ. In order to analyze the influence of these parameters on the blur kernel estimation, we collect 10 blurred images for tests. For each parameter, we carry out experiments with different parameter settings by varying one and fixing the others with the kernel similarity metric to measure the accuracy of estimated kernels. For parameter δ, we set its values from $10^{-5}$ to 0.01 with the step size of $5\times 10^{-4}$. Figure 10a demonstrates that blur kernels can be well estimated by a wide range of δ, i.e., within $\left[0.001,\;0.01\right]$. Similarly, we set the values of η and ρ from 0 to 2 with the increment of 0.1, and the values of γ and μ from 0 to 0.01 with the increase of $5\times 10^{-4}$. The experimental results of η and ρ parameters are shown in Figure 10b,c. Since γ=μ in the actual calculation process, the result is displayed by one curve, as shown in Figure 10d. The experimental results show that the proposed blur kernel estimation algorithm performs well with a wide range of parameter settings. In addition, when γ=μ=0, it can be seen from Figure 10d that the blur kernel estimation effect is extremely poor, which also proves the necessity of introducing Extreme Channels Prior to the blur kernel estimation model in this paper.

### 5.5. Comparison with the Deep Learning Method

**Figure 11:**
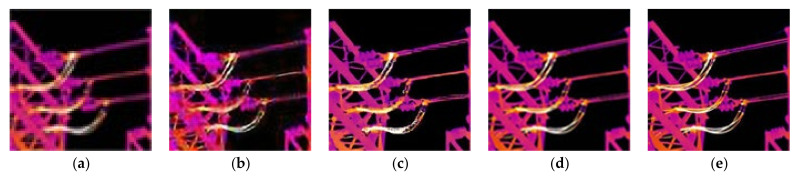
Comparison between deep learning and image reconstruction: (**a**) composite LR image; (**b**) inadequate training; (**c**) adequate training; (**d**) our method; (**e**) original image.

In order to reflect the superiority of this algorithm in practical applications, this section selects the advanced methods of deep learning algorithms [33] and our super-resolution reconstruction method for comparison experiments. At this stage, deep learning-based super-resolution algorithms require a large number of high-definition images as training samples. When training resources are insufficient, the performance of the method will decrease significantly. The algorithm in this paper can achieve high-quality image reconstruction without training samples. Figure 11 shows the comparison of the reconstruction results of the deep learning method with 500 and 2000 infrared images after training the model. It can be seen from Figure 11 that the deep learning algorithm has an obvious grid phenomenon and color distortion when the training data are insufficient. When the training data are sufficient, the contrast of some edge detail textures is slightly higher than that of our method. However, the result of the learning method contains a certain amount of false texture, which has a bad influence on infrared diagnosis. It can be seen that our method does not require training samples, and the reconstruction results are more accurate, so it has better practical application value in the electric power field.

## 6. Conclusions

In order to improve the quality of SR reconstructed images, so as to facilitate the status monitoring and fault analysis of power equipment, we propose a blind SR method of compressed sensing. Our method is divided into two parts: blur kernel estimation and non-blind SR reconstruction. For the blur kernel estimation part, we improved the basic SR model of compressed sensing and get the basic blur kernel estimation model. In order to improve the estimation accuracy of the blur kernel, we introduce Extreme Channels Prior based on the color characteristics of the infrared image. For the non-blind SR reconstruction method, we propose an adaptive non-blind SR reconstruction algorithm. It controls the intensity coefficient of the regular term adaptively during the reconstruction process to suppress the generation of artifact ringing and improve the quality of the reconstructed image. The above blur kernel estimation method and the non-blind SR method are combined to form our blind SR method. In the experimental part, we compare the two parts of the blind SR method with their corresponding existing classical methods to illustrate the superiority of the performance of our method. According to the experimental results, it can be seen that our method can estimate the blur kernel more accurately, which can complete the non-blind SR reconstruction of LR infrared images with higher quality. The HR infrared image reconstructed by our method has more detailed textures and better visual effects, which can provide better conditions for the application of power system infrared diagnosis. Under the current background of the power industry actively carrying out the construction of the IoT, our method provides a feasible way to reduce the hardware cost of its construction. We think it enjoys broad application prospects to use the mode of front-end using low-cost sensors to collect information and back-end using algorithms to recover the high-quality collected images. This method can effectively reduce the construction cost of IoT and the cost of data transmission and storage. Because the construction of power IoT is still in its infancy, we do not choose to use the data-driven method for infrared image super-resolution reconstruction. When the data acquisition and storage system become more standardized and mature in the future, we think that it will also be an interesting idea to train dictionaries according to the types of power equipment and update them online to achieve sparse representation of different images, which may be able to achieve a better reconstruction effect on the premise of ensuring the accuracy of image information.

## Figures and Tables

**Table 1:** The mean SSDE of each method for each BK on all 100 synthetic blurred images.

Image Number	Shao	Michaeli	Liang	Ours
BK1	0.0473	0.0485	0.0471	0.0464
BK2	0.0472	0.0438	0.0430	0.0419
BK3	0.0467	0.0444	0.0436	0.0423
BK4	0.0390	0.0379	0.0369	0.0351
BK5	0.0431	0.0429	0.0425	0.0415
BK6	0.0422	0.0406	0.0402	0.0395

**Table 2:** PSNR parameter values of reconstruction results of different methods.

Image Number	Key	Glasner	Dong	Zhao	Ours
1	24.144	25.556	25.789	28.345	29.137
2	27.752	29.622	32.439	32.597	33.778
3	24.372	25.733	27.128	28.135	30.015
4	25.719	27.352	28.095	30.420	31.582
5	26.483	27.303	27.406	29.248	31.738
6	27.851	29.844	29.978	31.581	33.833
7	23.438	24.324	28.360	29.587	30.974
8	20.518	24.047	24.322	26.452	28.039
9	24.804	26.015	28.222	30.799	31.129
10	24.173	24.405	25.642	26.825	29.384

**Table 3:** SSIM parameter values of reconstruction results of different methods.

Image Number	Key	Glasner	Dong	Zhao	Ours
1	0.787	0.839	0.873	0.884	0.904
2	0.881	0.920	0.954	0.948	0.967
3	0.883	0.903	0.959	0.949	0.973
4	0.832	0.864	0.877	0.927	0.931
5	0.828	0.856	0.865	0.899	0.925
6	0.818	0.827	0.849	0.858	0.905
7	0.798	0.833	0.888	0.902	0.931
8	0.797	0.829	0.866	0.898	0.908
9	0.800	0.826	0.846	0.918	0.922
10	0.899	0.919	0.926	0.931	0.952

## Data Availability

Not applicable.
